# The neutrophil/lymphocyte ratio as a predictor of successful conversion surgery for stage IV gastric cancer: a retrospective study

**DOI:** 10.1186/s12885-020-06884-4

**Published:** 2020-04-29

**Authors:** Naohiko Nakamura, Shinichi Kinami, Yasuto Tomita, Takashi Miyata, Hideto Fujita, Hiroyuki Takamura, Nobuhiko Ueda, Takeo Kosaka

**Affiliations:** Department of Surgical Oncology, Kanazawa Medical University Hospital, 1-1 Daigaku, Uchinada, Kahoku, Ishikawa 920-0293 Japan

**Keywords:** Gastric cancer, Conversion surgery, Neutrophil/lymphocyte ratio

## Abstract

**Background:**

Conversion surgery (CS) following a response to chemotherapy occasionally leads to prolonged survival in patients with stage IV gastric cancer (GC). This study aimed to evaluate the predictive value of the neutrophil/lymphocyte ratio (NLR) for the success of CS in patients with stage IV GC.

**Methods:**

We retrospectively analyzed data of 50 patients with stage IV GC who received systemic chemotherapy between January 2009 and December 2017 at the Kanazawa Medical University Hospital. The successful CS group included the patients who underwent R0 or R1 resection with CS, and the failed CS group included the patients who did not undergo CS after chemotherapy or those who, despite undergoing CS, had to additionally undergo R2 resection. Clinicopathological characteristics were examined in both groups. Univariate and multivariate analyses were performed to identify pretherapeutic parameters that were independently associated with the achievement of successful CS.

**Results:**

The number of patients in the successful and failed CS groups were 12 and 38, respectively. On univariate analysis, gender (*P* = 0.01), NLR (*P* = 0.003), albumin levels (*P* = 0.004), and absence of peritoneal metastasis (*P* = 0.004) were found to be significantly correlated with a successful CS. On multivariate analysis, NLR < 4 and absence of peritoneal metastasis were independently correlated with a successful CS (*P* = 0.02 and *P* = 0.002, respectively). In patients without peritoneal metastasis, successful CS rates in patients with NLR < 4 were significantly higher than those in patients with NLR ≥ 4 (61.1% vs. 10.0%, *P* = 0.005).

**Conclusions:**

The NLR was a significant independent predictor of the achievement of successful CS in stage IV GC patients, especially among the patients without peritoneal metastasis. Patients with a low NLR could have higher possibility of achieving successful CS.

## Background

Gastric cancer (GC) is the fifth most common malignancy and the second most common cause of cancer mortality worldwide [1]. Although early GC is largely a curable disease, advanced GC is still associated with poor survival. The curative treatment for advanced GC consists of gastrectomy with perioperative chemotherapy [2, 3], and chemotherapy remains the main therapeutic approach for stage IV GC [4]. Recently, a randomized, controlled trial of reduction surgery plus chemotherapy versus chemotherapy alone for stage IV GC (REGATTA trial) failed to show any efficacy for surgery [5]. In contrast, curative resection following systemic chemotherapy in initially unresectable GC is now called conversion surgery (CS). With the development and improved response of chemotherapy regimens, a number of CS has been proven to be successful in stage IV GC [6–10]. However, the significance of this approach and when it should be recommended for stage IV GC remains controversial. Furthermore, the heterogeneous presentation of stage IV GC could complicate the identification of the best therapeutic strategy for these advanced cases due to their different biological behaviors.

Recently, interest in the association between the neutrophil/lymphocyte ratio (NLR) and the clinical outcomes of upper gastrointestinal cancers has been growing worldwide. The NLR, that is easily measurable in a routine GC patient examination, is calculated as the neutrophil count divided by the lymphocyte count. A retrospective analysis of data on GC patients who underwent gastrectomy showed that a high NLR was associated with poor survival, tumor depth, and peritoneal metastasis [11]. In contrast, the association of NLR with clinical outcome of stage IV GC patients who underwent CS remains unclear. CS should be considered for stage IV GC patients who show good response to chemotherapy and for whom resection by CS provides a possible cure. However, it is sometimes difficult to predict success of CS before starting the chemotherapy course because stage IV GC patients have different metastasis patterns and a heterogeneous background. Thus, we considered that studying the NLR before chemotherapy could help predict a successful CS and construct the therapeutic strategy for stage IV GC patients. This study aimed to evaluate the clinicopathological characteristics of stage IV GC patients who underwent successful CS and the predictive value of the NLR in this context.

## Methods

### Patients

We retrospectively analyzed data on 50 patients with stage IV GC who underwent systemic chemotherapy at Kanazawa Medical University Hospital between January 2009 and December 2017. Based on the imaging studies or staging laparoscopy before treatment, the patients were clinically staged by the 15th edition of the Japanese Classification of Gastric Carcinoma [12] according to depth of tumor invasion (T), extent of lymph node metastasis (N), and distant metastasis (M). We divided the 50 patients into two groups: the successful CS group included the patients who underwent R0 or R1 resection by CS, and the failed CS group included the patients who could not undergo CS after chemotherapy or those that, despite undergoing CS, had to additionally undergo R2 resection. Informed consent was obtained from the patients by description. The Medicine Ethics Committee of Kanazawa Medical University approved this study.

### Treatments

The first-line chemotherapy treatments delivered were S-1/cisplatin, S-1/oxaliplatin, S-1/docetaxel, and capecitabine plus cisplatin with or without trastuzumab. Responses to chemotherapy were classified according to the Response Evaluation Criteria in Solid Tumors (RECIST) guidelines [13]. When patients who were initially regarded as unresectable responded well to chemotherapy, gastrectomy and/or metastasectomy were considered if R0 resection was possible. Gastrectomy with lymph node dissection was performed only in patients with distant metastasis who achieved complete response to chemotherapy. Postoperative adjuvant chemotherapy consisted on S-1 monotherapy or S-1 combined with another drug until recurrence, depending on the effectiveness of the surgery.

### Evaluations

Pre-treatment clinical data, such as gender, age, and body mass index, were collected from our hospital’s record. We extracted the results of the blood examination before the treatment, including white blood cell (WBC) count, the fraction of neutrophils and lymphocytes in the WBC differential, NLR, hemoglobin level, serum platelet count. We additionally analyzed levels of C-reactive protein (CRP), total protein (TP), albumin, cholinesterase, carcinoembryonic antigen (CEA), carbohydrate antigen 19–9 (CA 19–9), and carbohydrate antigen 125 (CA 125). Overall survival (OS) was considered from the date the chemotherapy treatment began until death caused by GC or other causes. In the case of patients who survived during our analysis, the date of the last follow-up was December 31, 2018.

### Statistical analysis

Data were expressed as n (%) or mean (± standard deviation). Continuous variables and categorical variables were compared using the Student’s t-test and the χ^2^ test, respectively. All *P*-values were two-sided, and differences with a *P*-value < 0.05 were considered as statistical significance. OS analysis was performed using the Kaplan-Meier method and results were examined using the log-rank test. A logistic regression model was used to identify clinical factors that were independently associated with a successful CS result. Variables that were associated with a successful CS result with *P* ≤ 0.05 in the univariate analysis were included in the multivariate analysis. We performed the multivariate receiver operating characteristic (ROC) curve analysis for the diagnostic performance of the NLR with regard to the successful CS. The JMP software version 8.0 (SAS Institute, Cary, NC, USA) was used for all statistical analyses.

## Results

### Patient characteristics

The number of patients in the successful and failed CS groups were 12 and 38, respectively. The patient characteristics before the treatment in both groups are shown in Table 1. In the successful CS group, the proportion of males was significantly higher than in the failed CS group. The successful CS group showed significantly lower NLR and a higher albumin level than the failed CS group. Although there was no significant difference, the proportion of patients who had abnormal levels of tumor markers tended to be higher in the failed CS group than in the successful CS group. With regard to the distant metastatic factors considered for stage IV diagnosis, peritoneal metastasis was significantly higher in the failed CS group compared with the successful CS group. In contrast, lymph node metastasis was higher in the successful CS group than in the failed CS group (Table 2). The proportion of patients in the successful and failed CS groups who underwent chemotherapy regimen combining more than two agents before surgery was 100 and 76.3%, respectively (*P* = 0.06).

**Table 1:** Patient characteristics in the successful and failed conversion surgery groups

	SC group (*n* = 12)	FC group (*n* = 38)	*P* value
Gender (male)	11 (91.7%)	21 (55.3%)	0.02
Age	69.7 (±3.2)	68.6 (±1.8)	0.78
BMI	21.9 (±1.3)	20.9 (±0.7)	0.53
White blood cell (/μl)	6593 (±473)	6047 (±266)	0.32
NLR	2.4 (±0.6)	4.4 (±0.4)	0.008
Hemoglobin (g/dl)	11.2 (±0.4)	11.4 (±0.6)	0.79
Platelet count (× 10^4^/μl)	31.3 (±3.6)	30.4 (±1.9)	0.81
CRP (mg/dl)	0.8 (±0.7)	1.3 (±0.4)	0.53
Total protein (g/dl)	6.8 (±0.2)	6.6 (±0.1)	0.38
Albumin (g/dl)	3.8 (±0.2)	3.4 (±0.1)	0.04
Cholinesterase (U/l)	230 (±27)	212 (±15)	0.55
CEA (< 5 ng/ml)	9 (75.0%)	20 (52.6%)	0.17
CA19–9 (< 37 U/ml)	10 (83.3%)	21 (55.3%)	0.08
CA125 (< 35 U/ml)	10 (83.3%)	34 (72.7%)	0.46

Values are in n (%) or mean (± standard deviation)

Abbreviations: *BMI* Body mass index, *NLR* neutrophil/lymphocyte ratio, *CRP* C-reactive protein, *CEA* carcinoembryonic antigen, *CA 19–9* carbohydrate antigen 19–9, *CA 125* carbohydrate antigen 125

**Table 2:** Distant metastatic factors for stage IV in the successful and failed conversion surgery groups

	SC group (*n* = 12)	FC group (*n* = 38)	*P* value
Peritoneal metastasis	0 (0%)	22 (57.9%)	0.0004
Lymph node metastasis	10 (83.3%)	12 (31.6%)	0.002
Liver metastasis	1 (8.3%)	0 (0%)	0.09
Cytological malignancy (+)	1 (8.3%)	2 (5.3%)	0.7

Values are in n (%)

### Overall survival analysis in the successful and failed conversion surgery groups

**Fig. 1:**
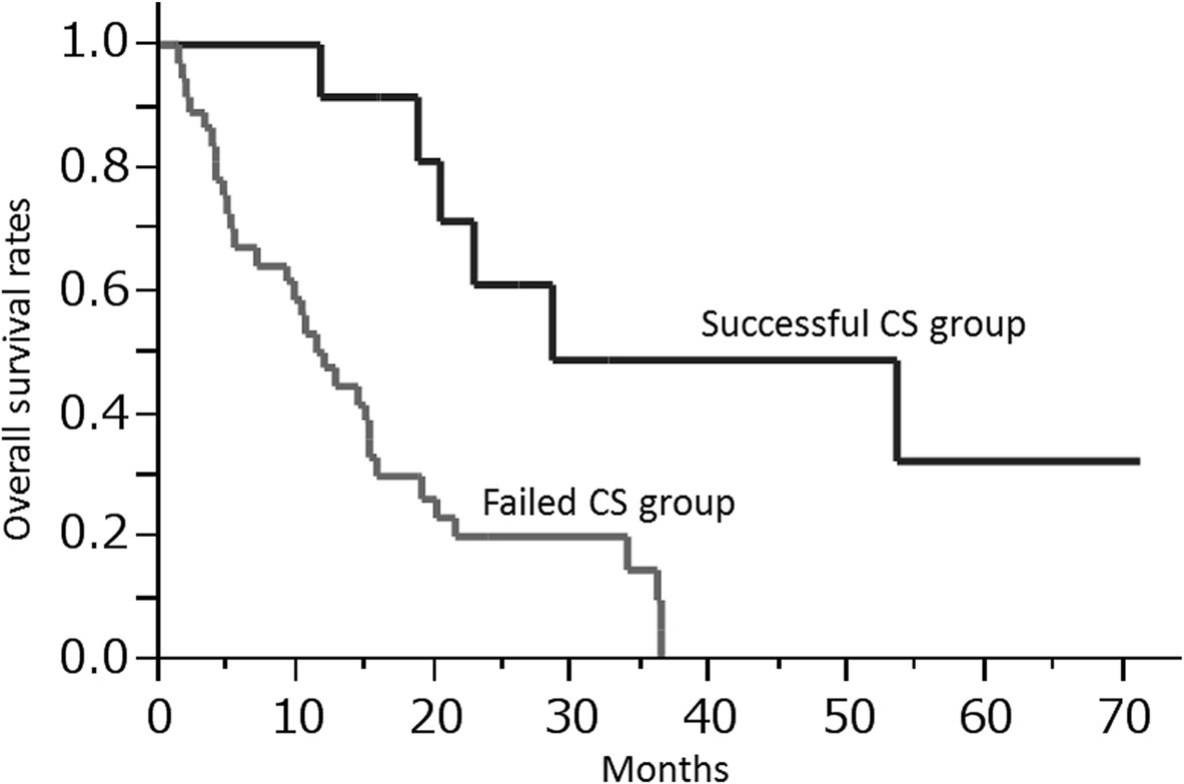
Overall survival rates in the successful and failed conversion surgery groups. The median survival rates were 28.5 and 11.9 months in the successful and failed CS groups, respectively (*P* = 0.0007 [log-rank])

The median survival rates were 28.5 and 11.9 months in the successful and failed CS groups, respectively (Fig. 1). The successful CS group had a significantly better prognosis after the treatment (*P* = 0.0007 [log-rank]).

### Correlation between pretherapeutic parameters and successful conversion surgery

The area under the ROC curve of the NLR between the successful CS and failed CS groups was 0.77. The best cutoff point of the NLR for distinguishing a successful result was 4.4. At this cutoff point, the sensitivity and specificity were 1.0 and 0.50, respectively. Therefore, we regarded the NLR cutoff point as 4.0 in the univariate and multivariate analyses. On univariate analysis, gender (male) (*P* = 0.01), NLR (*P* = 0.003), albumin levels (*P* = 0.004), and absence of peritoneal metastasis *(P* = 0.004) were significantly correlated with successful CS (Table 3). On multivariate analysis, NLR < 4 and absence of peritoneal metastasis were significantly correlated with successful CS (*P* = 0.02 and *P* = 0.002, respectively). In contrast, there was no significant difference in the predictive values of the tumor markers CEA, CA 19–9, and CA 125. Absence of peritoneal metastasis and a low NLR before treatment were independently associated with achievement of successful CS.

**Table 3:** Univariate and multivariate analyses to identify pretherapeutic predictors of successful conversion surgery

	Univariate analysis		Multivariate analysis	
	Odds ratio	*P* value	Odds ratio	*P* value
Age (< 75)	1.59	0.18		
Gender (male)	8.9	0.01	10.4	0.06
BMI (> 20)	1.62	0.51		
Hemoglobin (> 12 g/dl)	1.09	0.89		
White blood cell (> 6000/μl)	0.9	0.87		
NLR (< 4)	12.2	0.003	16	0.02
CRP (< 1 mg/dl)	1.55	0.6		
Total protein (> 6.5 g/dl)	1.96	0.35		
Albumin (> 3.5 g/dl)	8.57	0.004	1.3	0.82
Cholinesterase (> 200 U/l)	1.24	0.75		
CEA (< 5 ng/ml)	2.7	0.16		
CA19–9 (< 37 U/ml)	4.05	0.07		
CA125 (< 35 U/ml)	1.87	0.45		
Negative for peritoneal metastasis	18.4	0.004	21.9	0.002
Tumor differentiation (tub1–2)	1.16	0.87		

Abbreviations: *BMI* Body mass index, *NLR* neutrophil/lymphocyte ratio, *CRP* C-reactive protein, *CEA* carcinoembryonic antigen, *CA 19–9* carbohydrate antigen 19–9, *CA 125* carbohydrate antigen 125

### Successful conversion surgery rates according to the neutrophil/lymphocyte ratio in subgroup analysis

**Fig. 2:**
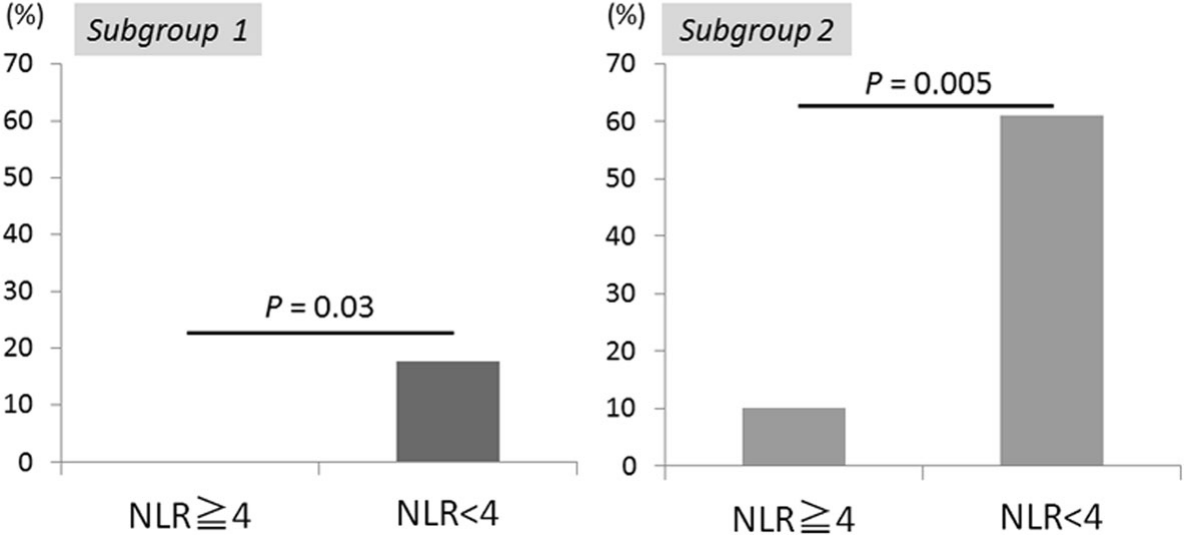
Successful conversion surgery rates in the subgroup analysis regarding the location of distant metastases. Subgroup 1: Patients with either single liver metastasis, para-aortic lymph node metastasis, or positive cytology were excluded (*N* = 33). Subgroup 2: Patients with no evidence of peritoneal metastasis were included (*N* = 28). In the subgroup 1, the successful CS rates were 0 and 17.7% in the patients with NLR ≥ 4 and NLR < 4, respectively (*P* = 0.03). In the subgroup 2, the successful CS rates were 10.0 and 61.1% in the patients with NLR ≥ 4 and NLR < 4, respectively (*P* = 0.005)

Based on the results in the multivariate analysis, we conducted a subgroup analysis regarding the distant metastatic types (Fig. 2). In the first subgroup analysis, we excluded patients with either single liver metastasis, para-aortic lymph node metastasis, or positive cytology. In the 33 patients included in this subgroup, the successful CS rates were 0 and 17.7% in the patients with NLR ≥ 4 and NLR < 4, respectively (*P* = 0.03). The second subgroup included 28 patients who had no evidence of peritoneal metastasis, and those with NLR < 4 had significantly higher successful CS rates than patients with NLR ≥ 4 (61.1% vs. 10.0%, respectively, *P* = 0.005). When peritoneal metastasis is not detected, stage IV patients with NLR < 4 could have higher potential for converting R0 or R1 resection after chemotherapy.

## Discussion

The first report of CS in GC took place in 1997 [14]. As chemotherapy progresses for advanced GC, CS following a response to chemotherapy occasionally leads to prolonged survival in patients with initially unresectable GC. One of the important indications for performing CS is whether the primary lesion and metastatic sites of GC could be curatively resected with this method. However, it is unclear what kinds of patients achieve a good response to chemotherapy and a successful CS. Thus, surrogate markers reflecting the heterogeneity in both clinical and oncological characteristics of stage IV GC patients are of great interest in order to better adjust the therapeutic strategy for these patients. In this study, we demonstrated that the NLR was independently associated with successful CS for stage IV GC and we evaluated the predictive value of the NLR for achievement of a successful CS, regardless of the location of the distant metastases.

Several studies have reported that CS for stage IV GC results in long-term survival in selected patients [6–8]. Our results showing the successful CS group had better prognosis than the failed CS group are in agreement with previous reports. It has also been demonstrated that non-invasive macroscopic type, higher differentiation, and absence of peritoneal dissemination were all favorable predictors of survival after CS [6]. In contrast, there are few reports analyzing the association between pretherapeutic laboratory parameters and achievement of successful CS. Yoshida et al. indicated new categories of classification for the patients with stage IV GC who may benefit from surgery after induction chemotherapy [15]. In this biological category, absence of macroscopic peritoneal metastasis is a very important factor for suggesting CS. We have confirmed that absence of peritoneal metastasis in the pretherapeutic setting independently correlated with successful CS. However, conventional imaging tests before treatment could limit the accurate detection of peritoneal metastasis [16, 17]. In addition, localized peritoneal metastasis sometimes disappears following chemotherapy. Deciding which patients would be good candidates for CS may be a difficult task by only taking into account the presence of peritoneal metastasis before treatment. On the other hand, we demonstrated that low NLR in the pretherapeutic setting was independently associated with successful CS with higher sensitivity. Most importantly, the subgroup analysis showed that NLR was associated with successful CS regardless of the characteristics of the distant metastasis. Thus, stage IV GC patients—especially those without peritoneal metastasis—who have low NLR have a higher potential for successful CS, and should undergo adequate chemotherapy and diligent assessment of their response to it aiming for conversion surgery.

The NLR is a simple index that could be calculated in a routine examination as the non-invasive biomarker of systemic inflammatory response [18, 19]. The elevated NLR have been previously reported to be related to the poor prognosis of GC, colorectal, and lung cancer [11, 18, 20]. Because the NLR is one of the parameters reflecting systemic inflammation, these previous results could be indicated there is a close relationship between inflammation and cancer progression. We have also previously shown that high NLR was associated with the presence of peritoneal metastasis during staging laparoscopy in patients with advanced GC [21]. GC cell migration and invasion were promoted by the interaction of neutrophils with tumor cells through a pathway involving interleukin 6 (IL-6) [22]. Therefore, an increased neutrophil count may reflect the condition of disease progression involving an upregulation of IL-6. In our analysis, 27.6% of patients with NLR < 4 showed complete or partial response to chemotherapy according to RECIST. In contrast, the proportion of patients who showed complete or partial response to chemotherapy was 14.3% in patients with NLR ≥ 4. The immune response of the host to tumors depends on the lymphocytes [23], and an increased number of neutrophils suppress the cytolytic activity of lymphocytes [24, 25]. Since, high NLR doe to an increased neutrophil count and a decreased lymphocytes counts may be related to the worse response to chemotherapy and failed CS in stage IV GC patients.

This study has certain limitations. It was a retrospective study performed at a single institution and the sample size was very small. Thus, to confirm the predictive value of the NLR for successful CS in stage IV GC, further work with a prospective cohort study in multiple institutions is warranted.

## Conclusions

The NLR was a significant independent predictor of the achievement of successful CS in stage IV GC patients. Patients with a low NLR could have a higher possibility of achieving curative resection by CS. Using the NLR in the clinical setting, we could further adjust the indication for CS and reconstruct the therapeutic strategy for stage IV GC patients.

## Data Availability

All data are available without restriction. Researchers can obtain data by contacting the corresponding author.

## Abbreviations

GC: Gastric cancer
CS: Conversion surgery
NLR: Neutrophil/lymphocyte ratio
WBC: White blood cell
CRP: C-reactive protein
CEA: Carcinoembryonic antigen
CA19–9: Carbohydrate antigen 19–9
CA125: Carbohydrate antigen 125
IL-6: Interleukin 6

## References

[1] Ferlay J, Soerjomataram I, Dikshit R, Eser S, Mathers C, Rebelo M (2015). Cancer incidence and mortality worldwide: sources, methods and major patterns in GLOBOCAN 2012. Int J Cancer.

[2] Cunnimgham D, Allum WH, Stenning SP, Thompson JN, Van de Velde CJ, Nicolson M (2006). MAGIC trial participants. Perioperative chemotherapy versus surgery alone for resectable gastroesophageal cancer. N Engl J Med.

[3] Brenkman HJ, Haverkamp L, Ruurda JP, van Hillegersberg R (2016). Worldwide practice in gastric cancer surgery. World J Gastroenterol.

[4] Sano T, Aiko T (2011). New Japanese classifications and treatment guidelines for gastric cancer: revision concepts and major revised points. Gastric Cancer.

[5] Fujitani K, Yang HK, Mizusawa J, Kim YW, Terashima M, Han SU (2016). REGATTA study investigators. Gastrectomy plus chemotherapy versus chemotherapy alone for advanced gastric cancer with a single non-curable factor (REGATTA): a phase 3, randomised controlled trial. Lancet Oncol.

[6] Einama T, Abe H, Shichi S, Matsui H, Kanazawa R, Shibuya K (2017). Long-term survival and prognosis associated with conversion surgery in patients with metastatic gastric cancer. Mol Clin Oncol.

[7] Fukuchi M, Ishiguro T, Ogata K, Suzuki O, Kumagai Y, Ishibashi K (2015). Prognostic role of conversion surgery for unresectable gastric cancer. Ann Surg Oncol.

[8] Kinoshita J, Fushida S, Tsukada T, Oyama K, Okamoto K, Makino I (2015). Efficacy of conversion gastrectomy following docetaxel, cisplatin, and S-1 therapy in potentially resectable stage IV gastric cancer. Eur J Surg Oncol.

[9] Morgagni P, Solaini L, Framarini M, Vittimberga G, Gardini A, Tringali D (2018). Conversion surgery for gastric cancer: a cohort study from a western center. Int J Surg.

[10] Beom SH, Choi YY, Baek SE, Li SX, Lim JS, Son T (2018). Multidisciplinary treatment for patients with stage IV gastric cancer: the role of conversion surgery following chemotherapy. BMC Cancer.

[11] Shimada H, Takiguchi N, Kainuma O, Soda H, Ikeda A, Cho A (2010). High preoperative neutrophil-lymphocyte ratio predicts poor survival in patients with gastric cancer. Gastric Cancer.

[12] Edge SB, Compton CC (2010). The American joint committee on Cancer: the 7th edition of the AJCC cancer staging manual and the future of TNM. Ann Surg Oncol.

[13] Eisenhauer EA, Therasse P, Bogaerts J, Schwartz LH, Sargent D, Ford R (2009). New response evaluation criteria in solid tumours: revised RECIST guideline (version 1.1). Eur J Cancer.

[14] Nakajima T, Ota K, Ishihara S, Oyama S, Nishi M, Ohashi Y (1997). Combined intensive chemotherapy and radical surgery for incurable gastric cancer. Ann Surg Oncol.

[15] Yoshida K, Yamaguchi K, Okumura N, Tanahashi T, Kodera Y (2016). Is conversion therapy possible in stage IV gastric cancer: the proposal of new biological categories of classification. Gastric Cancer.

[16] Burbidge S, Mahady K, Naik K (2013). The role of CT and staging laparoscopy in the staging of gastric cancer. Clin Radiol.

[17] Blackshaw GR, Barry JD, Edwards P, Allison MC, Thomas GV, Lewis WG (2003). Laparoscopy significantly improves the perceived preoperative stage of gastric cancer. Gastric Cancer.

[18] Walsh SR, Cook EJ, Goulder F, Justin TA, Keeling NJ (2005). Neutrophil-lymphocyte ratio as a prognostic factor in colorectal cancer. J Surg Oncol.

[19] Zahorec R (2001). Ratio of neutrophil to lymphocyte counts--rapid and simple parameter of systemic inflammation and stress in critically ill. Bratisl Lek Listy.

[20] Tomita M, Shimizu T, Ayabe T, Yonei A, Onitsuka T (2011). Preoperative neutrophil to lymphocyte ratio as a prognostic predictor after curative resection for non-small cell lung cancer. Anticancer Res.

[21] Nakamura N, Kinami S, Fujii Y, Miura S, Fujita J, Kaida D (2019). The neutrophil/lymphocyte ratio as a predictor of peritoneal metastasis during staging laparoscopy for advanced gastric cancer: a retrospective cohort analysis. World J Surg Oncol.

[22] Zhang W, Gu J, Chen J, Zhang P, Ji R, Qian H (2017). Interaction with neutrophils promotes gastric cancer cell migration and invasion by inducing epithelial-mesenchymal transition. Oncol Rep.

[23] Halazun KJ, Aldoori A, Malik HZ, Al-Mukhtar A, Prasad KR, Toogood GJ (2008). Elevated preoperative neutrophil to lymphocyte ratio predicts survival following hepatic resection for colorectal liver metastases. Eur J Surg Oncol.

[24] Petrie HT, Klassen LW, Kay HD (1985). Inhibition of human cytotoxic T lymphocyte activity in vitro by autologous peripheral blood granulocytes. J Immunol.

[25] el-Hag A, Clark RA (1987). Immunosuppression by activated human neutrophils. Dependence on the myeloperoxidase system. J Immunol.

